# Lower Eyelid Orbicularis Oculi Myocutaneous Flap: A Feasible Technique for Full Thickness Upper Eyelid Reconstruction

**DOI:** 10.29252/wjps.10.2.98

**Published:** 2021-05

**Authors:** Stefano Mori, Gianluca Di Monta, Ugo Marone, Gerardo Botti

**Affiliations:** 1 Istituto Nazionale dei Tumori Fondazione G. Pascale Via M. Semmola, 52 – 80131 Naples, Italy

**Keywords:** Basal cell carcinoma, Eyelid, Myocutaneous

## Abstract

Basal cell carcinoma (BCC) is the most frequent malignant eyelid tumor, followed by squamous cell carcinoma, sebaceous gland carcinoma and malignant melanoma. These eyelid malignancies represent main reason for eyelid reconstruction in ophthalmoplastic surgery which can be challenging. Lower eyelid orbicularis oculi myocutaneous flap was used for reconstructive purpose in four consecutive patients with a full thickness upper eyelid BCC. Digital photographs were taken at baseline, 1 month, 3 and 6 months after surgery to assess clinical outcome. Four patients underwent full thickness upper eyelid reconstruction with a lower eyelid orbicularis oculi myocutaneous flap after BCC radical resection with 3-mm safe margins. Histological subtypes showed tumor complete excision in all cases. No patient showed local recurrence at a mean 12 months follow-up. The high functional-esthetical success rate of the modified Hughes procedure corresponds with the beneficial results, which have been reported in previous publications. None of the treated patients complained about forced temporary closure of eyelid. In all four cases treated, aesthetic and functional outcome were satisfactory.

## INTRODUCTION

Approximately the 10% of skin cancers occur in the eyelid. The most frequency kind of malignancy is represented by the basal cell carcinoma (BCC) followed by squamous cell carcinoma, sebaceous gland carcinoma and malignant melanoma^1^. These eyelid neoplasms represent the main reason for eyelid reconstruction in ophthalmic plastic that can be challenging.

Aesthetic and functional reconstruction needs close attention to skin color, texture and thickness matching with respect to eyelid aesthetic units and symmetry. When possible, flaps are preferred to skin grafts^2^^,^^3^. Several flaps have been reported for eyelid reconstruction, based on different donor areas.

Treatment to be recommended depends on the patient health general state, histopathological subtype, location and size of tumor. Cryosurgery, photodynamic therapy and surgical radical resection are some of the existing approaches.

The classification of reconstructive techniques is based on anatomical area requiring treatment: upper eyelid, lower eyelid, inner or external canthus. In the last years, lower eyelid has been considered a good source of skin graft to reconstruct the skin of upper eyelid. In this manuscript, we described a procedure to reconstruct upper eyelid and periorbital region by means of lower eyelid skin and underlying orbicularis muscle as a myocutaneous flap^4^. 

The main procedure to reconstruct substantial horizontal full-thickness defects of lower eyelid is the Hughes procedure commonly used for: “low rate of complications, better functional and esthetical outcome, high patient satisfaction”^5^. 

The aim of this study was to describe our experience with modified Hughes procedure reporting functional and esthetical outcome and common complications^5^. 

## CASE PRESENTATION

**Scheme 1:**
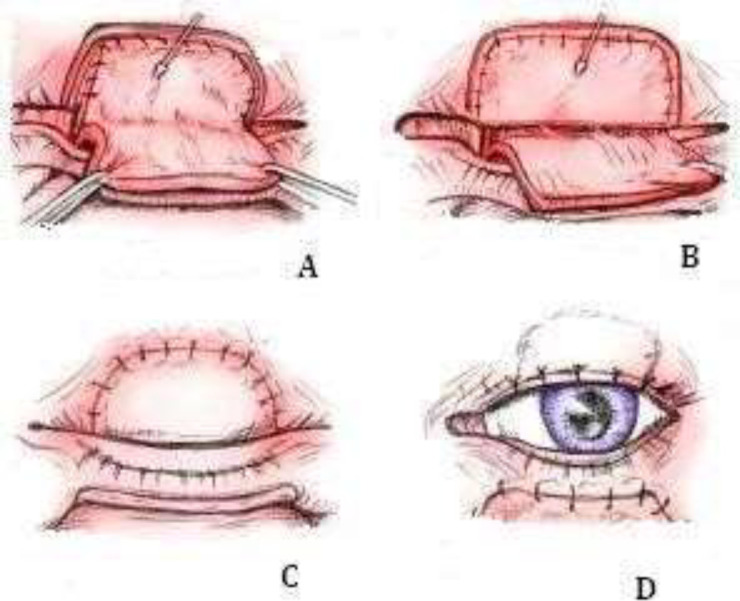
Superior eyelid reconstruction. A - B) Inferior eyelid miocutaneous flap moved into the defect of superior eyelid. C) De-epithelised pedicle flap was sutured tarsus to tarsus. D) Flap pedicle division

**Fig. 1:**
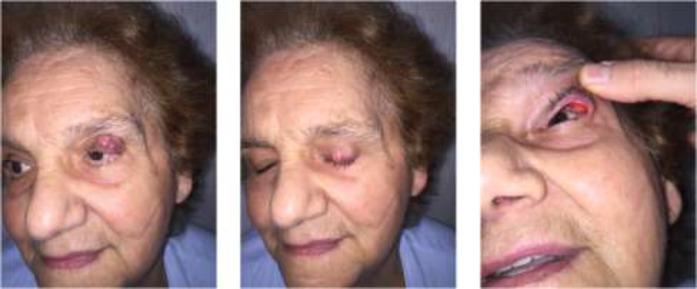
Pre-operative view of the patient

Lower eyelid orbicularis oculi myocutaneous flap was used for reconstructive purpose in four consecutive patients who presented to our Department with a full thickness upper eyelid BCC (Figure 1, Scheme 1). Digital photographs were taken at baseline, 1 month, 3 and 6 months after surgery to assess clinical outcome (Table 1). 

Each patient treated with this specific eyelid reconstruction signed the informed consent and the consent to treat personal data. The protocol was in accordance with the ethical guidelines of the 1975 Declaration of Helsinki; moreover, the local Ethics Committee approved the protocol.

**Table 1:** Patients data

**Patient**	**Gender**	**Age (yr)**	**Follow Up**
**1**	F	70	34 months
**2**	F	72	30 months
**3**	F	79	34 months
**4**	F	77	30 months

***Anatomy***

Upper and lower eyelids are constituted of several structures that can be grouped into anterior, middle, and posterior lamellae. Anterior lamella refers to the skin and eyelid orbicularis oculi muscle. The posterior lamella refers to the retractors, superior or inferior tarsal muscle, tarsus and conjunctiva. Just below the skin of the upper eyelid lies the orbicularis oculi, divided into orbital and palpebral portions^7^. The orbital portion is formed by the muscle fibers of the medial canthal tendon that have the function to close eyes tightly. Instead, palpebral portion is constituted by the semilunar muscle fibers that span medial and lateral canthal tendons. Capsulopalpebral fascia and inferior tarsal muscle form the lower lid retractors that attach to tarsal plate. Unlike levator aponeurosis of upper eyelid, capsulopalpebral fascia has few attachments to the skin and thus the fold of the lower eyelid is poorly formed. Although loss of levator of the upper lid tarsus can cause ptosis, loss of attachments of capsulopalpebral fascia to lower lid tarsus can cause rotational instability of lower eyelid^7^. 

***Technique***

**Fig. 2:**
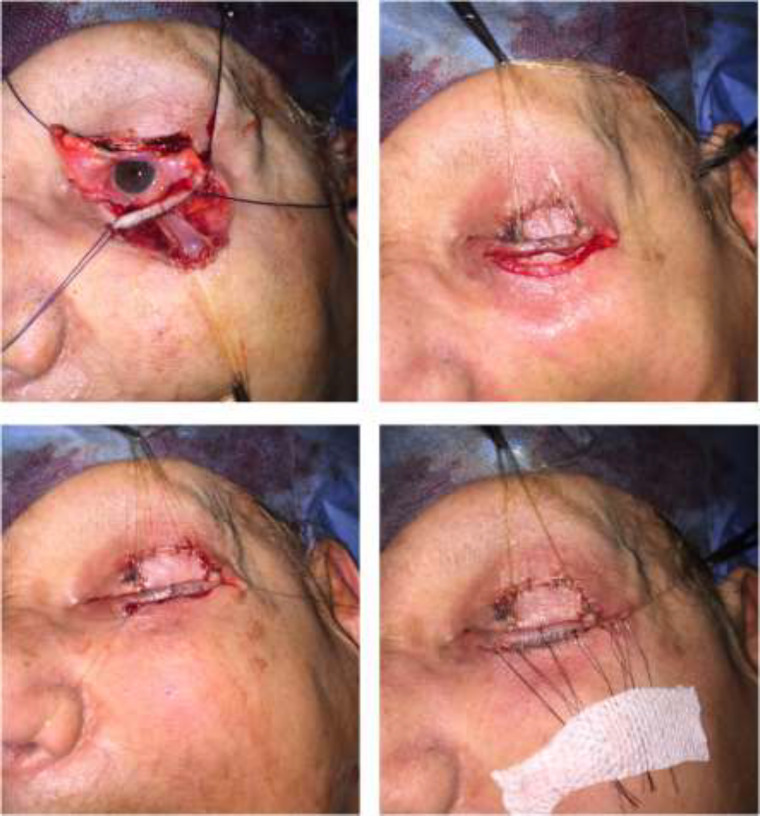
Intra-operative view

After expected defect was identified, lower eyelid orbicularis oculi muscle was detected, asking the patient to close his eyes tightly. A lower eyelid cutaneous island lying just above orbicularis oculi muscle was planned and shaped on upper eyelid defect, following radical resection of the tumor. Surgery was performed under local anesthesia with lidocaine with adrenaline 1:100,000. After excision was performed, maximum length of defect was measured and skin incision at the periphery of lower eyelid flap, dissection was carried out in a submuscular plane, below attachments of capsulopalpebral fascia to lower lid tarsus (Figure 2). The de-epithelised pedicle was buried deep beneath lower lid tarsus and flap was sutured tarsus to tarsus into defect using absorbable sutures. Flap pedicle division was undertaken under local anesthesia 4 wk after procedure^1^^,^^2^. 

**Fig. 3:**
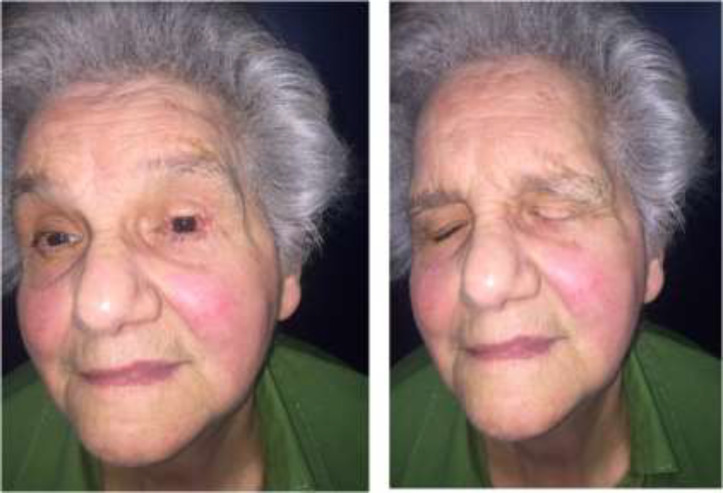
Six months post-operative view of the patient

## DISCUSSION 

Four patients underwent full thickness upper eyelid reconstruction with a lower eyelid orbicularis oculi myocutaneous flap after BCC radical resection with 3-mm safe margins. Maximum diameter of full thickness upper eyelid defect, reconstructed with the myocutaneous flap, was 2x1 cm. Flaps survived in all reported cases, even though all of them were reddish in color during the first 3 d after surgery, probably due to initial poor venous drainage and slight swelling. Although remarkable flap width, eyelids closed rightly and performed reconstruction was effective for skin texture and color match. Histological subtypes showed tumor whole excision in all four cases. No patient showed local recurrence at a mean follow-up of 32 months. 

Reconstruction of eyelid and periorbital area is difficult cause of quality of tissues in this area. Flaps generally used for reconstruction are thick and stiff. Medial and lateral canthal regions contribute to both normal function of eye and first impression of a person. Defects in this area can be caused by either trauma or skin malignancies, such as BCC and 14% of cases of basal cell carcinoma occur in the periorbital area^8^^,^^9^. Elderly population is especially vulnerable to skin tumors of face, due to cumulative effects of ultraviolet radiation exposure and lack of education about importance of sunscreen use^10^. Although these skin tumors are easily detected at an early stage, in locally advanced tumors, resection margins need to be extended to orbicularis oculi muscle layer. Completely set of good surgical planning, anatomical knowledge and adequate reconstruction technique indication produce low complication rate of ectropion, epiphora, edema, hemorrhage. This report aimed to demonstrate another option of upper eyelid reconstruction, as well as illustrating its steps regarding surgical planning. Surgical technique needs to be tailored to each patient and to subtype of BCC. Reconstructive techniques with free grafts and flaps yield excellent esthetic and functional results in the orbital region if good conditions for graft harvesting are respected accurate hemostasis of the receiving bed by moderate use of diathermy; careful suture of the edges; application of a compressive roll of gauze for at least 4 days^11^. Furthermore, it is recommended that the graft surface be 1/3 wider than that of the area to be covered, especially in cases of scar retraction. We used a modified Landolt-Hughes technique for reconstruction of upper eyelid defects with tarsal-conjunctival myocutaneous flap from lower eyelid in all cases of total -sub-total loss of upper eyelid resulting to removal of BCC. Modified Hughes is a full-thickness procedure for upper eyelid defect involving >50% of horizontal lid length ^12^^,^^13^. 

In all cases treated, reconstruction was performed by carving a tarsal-conjunctival flap from lower eyelid of an appropriate size to the loss of substance. A high aesthetic-functional success rate of the modified Hughes procedure corresponds to beneficial results, reported in previous publications. None of the treated patients complained about forced temporary closure of eyelid. The satisfaction degree of the operation outcome was very high in all patients treated (Figure 3).

## CONCLUSION

This procedure can be considered an important technique for repairing large full-thickness upper eyelid defects involving up to 100% of the horizontal lid length showing optimal results. In all four cases treated, aesthetic and functional outcome were satisfactory.

## CONFLICT OF INTEREST

The authors declare that they have no competing interests.
